# Dietary restriction mitigates phenotypes induced by traumatic brain injury (TBI) in female *Drosophila*

**DOI:** 10.17912/micropub.biology.001364

**Published:** 2024-11-19

**Authors:** Rebecca Ray, Rebecca Delventhal

**Affiliations:** 1 Department of Neuroscience, Yale University; 2 Department of Biology, Lake Forest College

## Abstract

TBI occurs when sudden trauma to the head causes damage to the brain, leading to long-term health problems. Many features of TBI can be replicated in *Drosophila* , making them an ideal model. Previous research on male flies showed that TBI decreases lifespan and locomotion, both of which were ameliorated by dietary restriction (DR). Considering female flies are known to be more responsive to DR, we examined whether DR ameliorates the effect of TBI in females. We found DR significantly extended lifespan and improved climbing ability at 2 weeks post-TBI, consistent with prior results in males.

**Figure 1. Dietary restriction (DR) mitigates phenotypes induced by TBI in female Drosophila:**
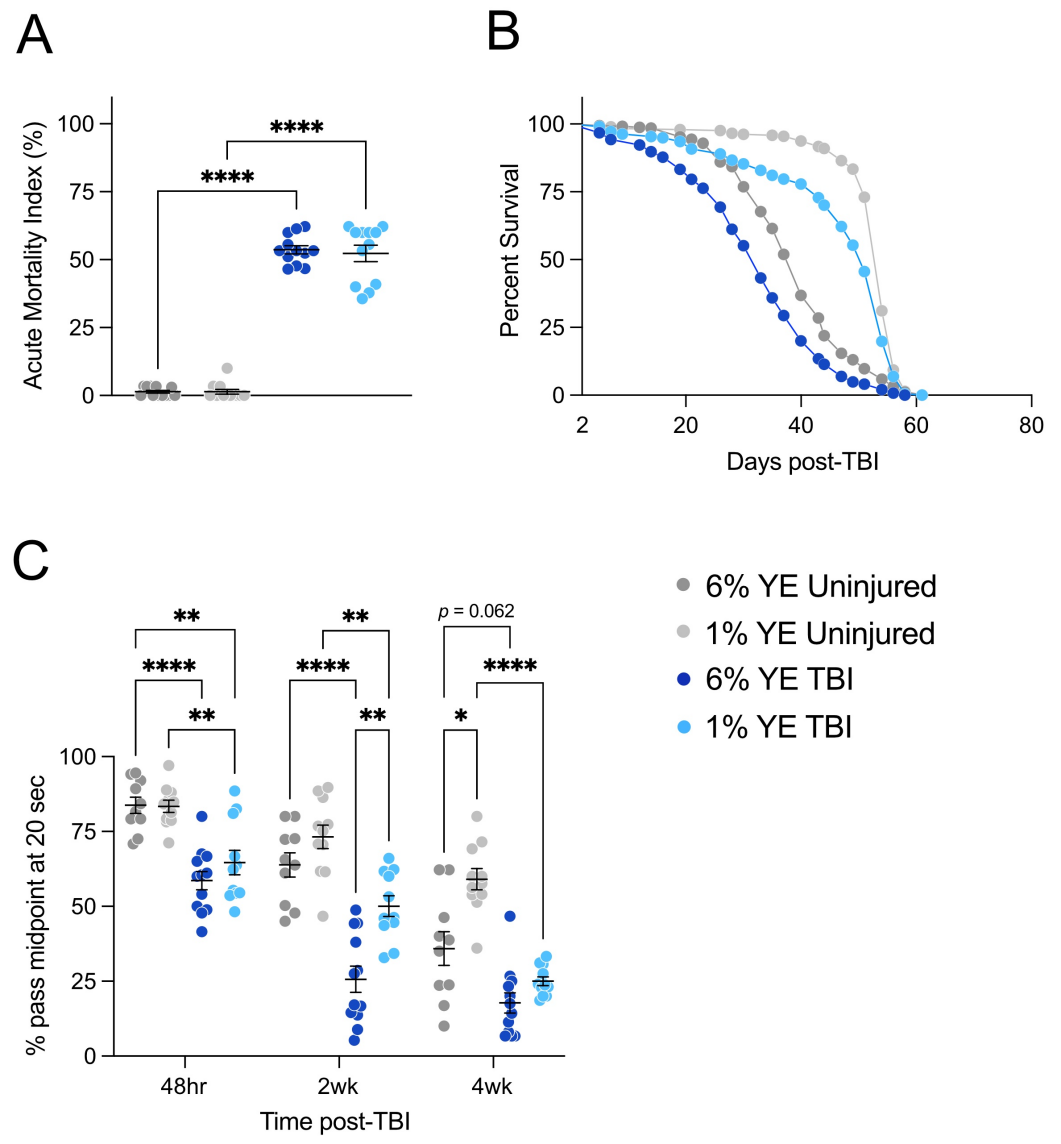
Uninjured controls fed non-DR 6% yeast extract (YE) food are marked in dark gray, uninjured controls fed DR 1% YE food are marked in light gray, injured (TBI) flies fed non-DR 6% YE food are marked in dark blue, and TBI flies fed DR 1% YE food are marked in light blue. **A.** Acute mortality of flies used in lifespan assay calculated at 24-48 hours post-TBI. TBI flies had significantly higher acute mortality than uninjured controls within both 6% YE ( *p < 0.0001* ) and 1% YE conditions ( *p < 0.0001* ). No significant difference in acute mortality was found between 6% YE TBI flies and 1% YE TBI flies. Similarly, no significant difference was found between 6% YE uninjured controls and 1% YE uninjured controls. Acute mortality was analyzed using a two-way ANOVA with Uncorrected Fisher’s LSD test for post-hoc analysis; sample size was 12 vials of ~35-45 flies each. **B.** Lifespans of surviving flies 48 hours post-TBI. TBI flies fed 1% YE food had a significantly longer lifespan than those fed 6% YE food, *p < 0.0001* . Similarly, uninjured control flies on 1% YE food lived significantly longer than those on 6% YE food, *p < 0.0001* . Within both 1% YE and 6% YE diet conditions, uninjured control flies had significantly longer lifespans than TBI flies, *p < 0.0001* . Lifespans were analyzed using a Mantel-Cox long-rank test; sample size was 217-337 flies per condition. **C.** Climbing ability of the same cohort of flies at 48 hours, 2 weeks, and 4 weeks post-TBI, as measured by percent of flies past the midline by 20 seconds. At 48 hours, there was no significant difference in climbing ability across food conditions, however, within both diet types TBI flies climbed significantly worse than uninjured controls, *p < 0.005* . At 2 weeks, 1% YE TBI flies had significantly better climbing ability than 6% YE TBI flies ( *p < 0.005* ), though at 4 weeks this difference lost significance. Conversely, climbing ability was not significantly different between 6% and 1% YE uninjured controls at 2 weeks, but at 4 weeks, 1% uninjured controls climbed significantly better, *p < 0.05* . Notably, while we found that 1% YE TBI flies climb significantly worse than 6% YE uninjured controls at 48 hours ( *p <* 0.01), there was no significant difference at 2 and 4 weeks, suggesting a compensatory role of DR. Climbing ability was analyzed using a two-way repeated measures ANOVA with Tukey’s multiple comparisons test for post-hoc analysis; sample size was 10-12 vials of 15-30 flies per condition.

## Description

Traumatic brain injury (TBI) poses a great threat to global health, with almost 70 million individuals experiencing a TBI every year worldwide (Dewan et al., 2018) . TBI can cause a range of acute and chronic neurological problems and has been associated with many long-term health complications, including cognitive and memory deficits and an increased risk of developing neurodegenerative diseases, such as Parkinson’s and Alzheimer’s Disease (Bower et al., 2003; Fleminger, 2003; Guskiewicz et al., 2005; Mckee & Daneshvar, 2015) . TBI can also disrupt important homeostatic mechanisms, such as metabolism (Lai et al., 2022; Yoshino et al., 1991) . Due to the high energy demands of the brain, cerebral metabolic dysfunction can be especially damaging and lead to cognitive impairment, neurodegeneration, and the development of neurodegenerative disease (Boveris & Navarro, 2008; Xu et al., 2016; Yan et al., 2020) .

*Drosophila melanogaster* has long been used as a model to study human diseases, and previous studies have established it as a model to study TBI (Barekat et al., 2016; Behnke et al., 2021; Katzenberger et al., 2013) . In this study, we used the High-Impact Trauma (HIT) device developed by Katzenberger et al. (2013) to induce a full-body TBI to flies. Previous studies have shown that flies given a TBI with the HIT device display similar pathology to that of human TBI patients, including acute mortality, shortened lifespan, impaired locomotor ability, neurodegeneration, protein aggregation, and activation of the innate immune pathway (Corrigan et al., 2014; Delventhal et al., 2022; Friedan et al., 2015; Katzenberger et al., 2013) .

In both humans and flies, TBI is known to induce phenotypes characteristic of aging, such as locomotor decline, neurodegeneration, and metabolic dysfunction. Dietary restriction (DR) is a well-established method to mitigate many consequences of aging in multiple organisms, including *Drosophila* (Kapahi et al., 2017) . DR is defined as restriction of one or more dietary nutrients without causing malnutrition (Kapahi et al., 2017; Partridge et al., 2005) . Considering the parallels between TBI pathology and aging, and the impact of DR on aging phenotypes, we previously looked at the impact of DR on TBI outcomes in male *Drosophila* (Delventhal et al., 2022) . We found that restricting the flies’ protein intake partly ameliorated the effect of TBI on lifespan and locomotion, as measured by climbing ability (Delventhal et al., 2022) . Injured male flies on DR lived significantly longer and had significantly better climbing ability two weeks post-TBI than injured flies fed normal food. Such results indicate that DR is capable of mitigating some TBI outcomes and may help with injury recovery.

However, studies have shown evidence to suggest a sex-dimorphic effect of DR on lifespan in *Drosophila* , whereby females have a larger lifespan increase than males (Magwere et al., 2004; Regan et al., 2016) . One study reported that female flies on DR not only had a greater increase in lifespan than males, but females also required a smaller magnitude of restriction from a normal diet to obtain a maximized lifespan (Magwere et al., 2004) . Such evidence suggests that female flies have a more robust response to DR than males and that DR may be more influential to female physiology. Considering this and the effect of DR on TBI outcomes in male *Drosophila* found by Delventhal et al. (2022), we wanted to examine the effect of DR on TBI outcomes in female *Drosophila* .

In this study, we delivered a TBI to 5-7 day old, mated female *Canton-S* (CS) flies and compared acute and long-term outcomes between flies fed normal food containing 6% yeast extract (6% YE) and DR food containing 1% yeast extract (1% YE). We used an adapted version of the HIT device (Kuklinski et al., 2025) to deliver the TBI and measured acute mortality, lifespan, and climbing ability at multiple timepoints following the injury. While there was a significant increase in acute mortality due to TBI, as expected, we found no significant effect of DR on the number of flies that died acutely (within 48 hours) after injury ( Figure 1A ). This is perhaps unsurprising as the flies are only placed on DR food as 1-3 day old adults, a few days prior to the TBI. We did find that 1% YE TBI flies had significantly increased lifespan compared to 6% YE TBI flies ( Figure 1B ). A similar lifespan extending effect of DR was also found in uninjured control flies, which is consistent with previous findings (Kapahi et al., 2017; Partridge et al., 2005) . Strikingly, TBI flies on DR food (1% YE) lived significantly longer than uninjured control flies on regular food (6% YE). When examining locomotor ability, we found that, as expected, within both diet types and across all time points, a significantly lower percentage of TBI flies climbed past the midpoint of the vial within 20 seconds than uninjured controls, indicating significantly worse locomotor function ( Figure 1C ). At 48 hours post-TBI, flies fed 1% YE food exhibited no difference in climbing ability compared to flies fed 6% YE food in both injury conditions. At 2 weeks post-TBI, TBI flies climbed significantly better when fed 1% YE food compared to 6% YE normal food, indicating DR led to a significant increase in locomotor function among injured flies. However, there was not a significant difference between these groups at 4 weeks post-TBI. Uninjured controls fed 1% YE food did exhibit significantly better climbing ability than uninjured controls fed 6% YE food at 4 weeks post-TBI. Interestingly, while TBI flies fed 1% food climbed significantly worse than uninjured controls fed 6% YE food at 48 hours post-TBI, at 2 and 4 weeks post-TBI they display equivalent locomotor ability. This suggests that DR may compensate for the TBI-induced decline in locomotor ability long term.

The ameliorating effect of DR on TBI-induced lifespan and locomotor phenotypes we observed in female *Drosophila* is consistent with prior findings in males (Delventhal et al., 2022) . Interestingly, the extent to which DR mitigated TBI outcomes in female flies appears qualitatively greater than in male flies, though direct statistical comparison would be inappropriate given differences in data collection. The DR-mediated increase in lifespan of both TBI and uninjured control flies was larger in magnitude in females (TBI = 55%, control = 35%) than in males (TBI = 14%, control = 14%, Delventhal et al., 2022). Similarly, the DR-mediated increase in climbing ability at 2 weeks post-TBI was greater in female flies (95%) than in males (40%, Delventhal et al., 2022). Additionally, the equivalent climbing ability seen between female uninjured controls fed 6% YE food and TBI flies fed 1% YE food at 2 and 4 weeks post-TBI was not seen previously in males (Delventhal et al., 2022) . This qualitative comparison of our findings to that of Delventhal et al. (2022) raises the question of whether DR has a greater ameliorating effect on TBI outcomes in females than in males, highlighting the importance of performing direct, simultaneous comparisons of males and females to draw conclusions about sex-specific responses.

Furthermore, while we found no evidence of an effect of protein restriction on TBI-induced acute mortality in this study, other studies have suggested that manipulation of other dietary components can affect acute mortality. One study showed injured flies recovered onto food with a high sugar content had significantly higher mortality rates 24 hours after injury compared to injured flies fed a standard formula during recovery (Katzenberger et al., 2015) . In another study, flies’ acute mortality increased in a dose-dependent manner when recovered onto diets high in carbohydrates or high in both carbohydrates and protein (Blommer et al., 2021) .

Considering the varying outcomes of TBI under different dietary manipulations, examining the effect of restriction of other dietary components, such as sugar or fat, could yield insights into the pathways involved in TBI recovery. Investigating whether the effect of diet on TBI-induced phenotypes is sex-specific would also be vital as previous studies have shown that altering sugar intake can have sex-specific impacts on physiology, lifespan, and locomotion (Baenas & Wagner, 2022; Millington et al., 2022) . Additionally, while we examined the effect of post-eclosion, lifelong DR exposure on TBI outcomes, it would be important to examine whether changing the timing or duration of DR exposure alters its effect on TBI outcomes. Shorter adult exposure may offer insight into the critical window and required duration of DR treatment to see maximum ameliorating effects. Also in this study, we exclusively examined mated females, but it remains to be seen the impact that mating status has on DR’s effect on TBI outcomes or on TBI outcomes themselves. Mating has an immense impact on the physiology, lifespan, and metabolism of female flies (Kapahi et al., 2017; Landis et al., 2020) , potentially altering TBI pathology and recovery in females.

Overall, we found that DR ameliorates TBI-induced phenotypes in female *Drosophila* by extending lifespan and mitigating locomotor decline. Our findings help further our understanding of how DR is implicated in TBI outcomes and may offer new opportunities for TBI recovery and treatment strategies.

## Methods

*Drosophila strains and husbandry*

Female *Canton-S* (CS) flies (gift from J. Carlson) were used in all lifespan and locomotor assays. All flies were reared and collected on glucose medium (Archon Scientific) and housed in incubators (Percival Scientific) at 25°C with a 12h:12h light:dark diurnal cycle. Male and female flies were permitted to mate for up to 72 hours post-eclosion before being separated, after which only female flies were kept and placed on dietary restriction (DR) food. Flies were transferred to fresh DR food every 48-72 hours.

*Food preparation*

Food was prepared using the following ingredients: 1% agar (Genesee Scientific, Cat # 66-103), 1% propionic acid (Sigma, #402907), 1.6% of a 10% Tegosept (Apex, Cat # 20-258) solution in ethanol, 2% sucrose (Genesee Scientific Cargill Sucrose, Cat # 62-112), 4% dextrose (Genesee Scientific Tate & Lyle 457 Dextrose, Cat # 62-113), and either 6% yeast extract (YE) (Thermo Fisher Bacto Yeast Extract, Ref # 212720) for standard food or 1% YE for the dietary restriction condition. After preparation, 2mL of food was pipetted into empty fly vials and left to dry under a fan until no condensation was present.

*Traumatic brain injury delivery and conditions*

TBI delivery was conducted using an adapted version of the high-impact trauma (HIT) device developed by Katzenberger et al. (2013). The adapted device consists of a wooden frame with a metal spring attached, to which a plastic, shatter-resistant vial is tightly fitted to one end using Velcro tape. At rest, the vial sits on a foam pad that is level with the bottom of the wooden frame. Each vial is loaded with 40-45 flies and attached to the end of the spring. Using a handheld quick release archery trigger, a string is used to pull the spring back to a specified deflection angle of 85°, determined by a fixed metal rod. Once the vial is in place, the quick release trigger is opened, releasing the spring, and the vial impacts the pad, delivering one strike.

Experimental groups for injury conditions included TBI and uninjured controls. Flies were collected together from the same cohort of flies and were separated into either condition 24-72 hours post-eclosion. At 5-7 days of age, flies in the TBI injury condition were given a single, severe injury consisting of four strikes at an 85° angle spaced 5 minutes apart. After TBI delivery, flies were transferred to fresh food vials positioned horizontally and allowed to recover in the incubator for 24-48 hours. Uninjured control flies of the same age were treated the same as flies in the TBI condition, including being placed in HIT vials and transferred to recovery vials, with the only difference being the delivery of four HIT strikes. Flies in either injury condition were not anesthetized using CO _2_ within 24 hours of being placed in HIT vials.

*Acute mortality index*

The acute mortality index was calculated by dividing the number of flies dead in each vial 24-48 hours after TBI delivery by the total number of flies in the vial and converting to a percentage. Dead flies were excluded from subsequent experiments, and the remaining flies were maintained by transferring to fresh food every 48-72 hours. Acute mortality was always calculated after TBI was delivered, regardless of the type of assay or experiment that was to follow.

*Lifespan assay*

Starting 48 hours after TBI delivery, vials containing 15-30 surviving flies were transferred to new vials containing fresh food medium every 2-3 days. During each transfer, the number of dead flies was recorded.

*Locomotor assay*

Vials containing 10-30 flies were transferred to empty vials without the use of CO _2_ anesthesia. Another empty vial was secured atop using transparent tape to create a ~20 cm high climbing vial. Flies were allowed to acclimate to the climbing vials placed on their sides for 10 minutes prior to the assay. Then, 5-7 climbing vials at a time were secured vertically in a clear plastic apparatus within which humidity and temperature were maintained between 20-23 °C and 40-60% relative humidity. If the initial relative humidity was below 45%, a damp paper towel was placed inside the apparatus to raise the humidity. The apparatus was then tapped down firmly against a padded surface at least 5 times to gather the flies at the bottom of each vial. Flies were allowed to climb for 75 seconds before being tapped down again. The apparatus was tapped down a total of 5 times over 6 minutes. Each experiment was recorded for later analysis. Afterwards, flies were briefly anesthetized with CO _2_ to count the total number of flies in each climbing vial. Flies were then transferred to new vials containing fresh food medium. Locomotor activity of the same cohort of flies was measured at 48 hours, 2 weeks, and 4 weeks after injury. Video recordings of each assay were manually analyzed to count the number of flies that climbed past the halfway point on the vial, about 10 cm, in 20 seconds. Locomotor ability was quantified by calculating the percentage of flies that were at the halfway point or above (pass rate), averaged across five trials for each vial.

*Statistical analyses*

Statistical analyses were performed by GraphPad Prism (version 10.2.1). Acute mortality index was analyzed using a two-way ANOVA with an Uncorrected Fisher’s LSD test for post-hoc analysis. Lifespan data were analyzed with a Mantel-Cox log-rank test. Climbing data were analyzed using a two-way repeated measures ANOVA with a Tukey’s multiple comparisons test for post-hoc analysis.
